# Intrauterine Growth Restriction Promotes Postnatal Airway Hyperresponsiveness Independent of Allergic Disease

**DOI:** 10.3389/fmed.2021.674324

**Published:** 2021-05-31

**Authors:** Jack O. Kalotas, Carolyn J. Wang, Peter B. Noble, Kimberley C. W. Wang

**Affiliations:** ^1^School of Human Sciences, The University of Western Australia, Crawley, WA, Australia; ^2^Telethon Kids Institute, The University of Western Australia, Nedlands, WA, Australia

**Keywords:** airway hyperresponsiveness, allergy, intrauterine growth restriction, asthma, lung function

## Abstract

**Introduction:** Intrauterine growth restriction (IUGR) is associated with asthma. Murine models of IUGR have altered airway responsiveness in the absence of any inflammatory exposure. Given that a primary feature of asthma is airway inflammation, IUGR-affected individuals may develop more substantial respiratory impairment if subsequently exposed to an allergen. This study used a maternal hypoxia-induced mouse model of IUGR to determine the combined effects of IUGR and allergy on airway responsiveness.

**Methods:** Pregnant BALB/c mice were housed under hypoxic conditions (10.5% O_2_) from gestational day (GD) 11-GD 17.5 (IUGR group; term = GD 21). Following hypoxic exposure, mice were returned to a normoxic environment (21% O_2_). A second group of pregnant mice were housed under normoxic conditions throughout pregnancy (Control). All offspring were sensitized to ovalbumin (OVA) and assigned to one of four treatment groups: Control – normoxic and saline challenge; IUGR – hypoxic and saline challenge; Allergy – normoxic and OVA challenge; and IUGR + Allergy – hypoxic and OVA challenge. At 8 weeks of age, and 24 h post-aerosol challenge, mice were tracheostomised for methacholine challenge and assessment of lung mechanics by the forced oscillation technique, and lungs subsequently fixed for morphometry.

**Results:** IUGR offspring were lighter than Control at birth and in adulthood. Both Allergy and IUGR independently increased airway resistance after methacholine challenge. The IUGR group also exhibited an exaggerated increase in tissue damping and elastance after methacholine challenge compared with Control. However, there was no incremental effect on airway responsiveness in the combined IUGR + Allergy group. There was no impact of IUGR or Allergy on airway structure and no effect of sex on any outcome.

**Conclusion:** IUGR and aeroallergen independently increased bronchoconstrictor response, but when combined the pathophysiology was not worsened. Findings suggest that an association between IUGR and asthma is mediated by baseline airway responsiveness rather than susceptibility to allergen.

## Introduction

Asthma is an obstructive airway disease that affects patient quality of life, manifesting as episodes of breathing difficulties. Airway hyperresponsiveness (AHR), a major functional impairment in asthma, results in disproportionate airway narrowing that produces airflow limitation (1). There are numerous potential causes of AHR. A relationship between AHR and allergy has been established; inflammation, orchestrated by T-helper 2 (Th2) cells, results in the release of bronchoactive mediators including histamine, leukotriene B4, prostaglandin D2 and cytokines along with the recruitment of immune cells (2, 3) which mediate excessive airway constriction. “Airway remodeling” that is either independent or co-dependent on inflammation (4), is also associated with AHR. Airway remodeling is a change in the structure (mass, thickness, or volume) of the airway wall (5), exerting a multitude of effects, including increased airway smooth muscle (ASM) force production (1), and reduced and more variable airway caliber (1, 6), all of which at least contribute to the onset of AHR.

The above changes to airway structure-function in asthma have conventionally been attributed to environmental exposures (e.g., allergic stimuli) accumulated through postnatal life. An alternative proposal is that airway abnormalities are the result of a developmental disorder and we particularly note the association between intrauterine growth restriction (IUGR) and asthma (7). After establishing a mouse model of hypoxia-induced IUGR, we demonstrated airway hyperresponsiveness in female offspring and hyporesponsiveness in males (8). Functional changes after IUGR were not associated with airway remodeling (8, 9), rather our data implicated a shift in inflammatory phenotype; an increase in macrophages in the bronchial alveolar lavage (BAL) fluid from both male and female offspring with males also demonstrating an increase in interleukin (IL)-2, IL-13, and eotaxin (10). Importantly, this shift in inflammatory phenotype was the result of a prenatal disruption that persisted into adult life and occurred without exposure to typical environmental triggers (10). Together these observations suggest that developmental changes in airway responsiveness that occur concomitantly with inflammation will alter the susceptibility to environmental influences and subsequent airway disease.

The present study was therefore principally focused on the evolution of AHR in asthma, which as discussed is impacted by structural and inflammatory pathologies and potentially developmental programming. We specifically examined the interaction between IUGR and allergy and hypothesized that persistent biological changes after IUGR worsens the response to allergy and this manifests as more severe bronchoconstriction to contractile stimulation i.e., AHR. To address this study hypothesis, we used our established mouse model of IUGR and exposed both male and female offspring to ovalbumin (OVA) sensitization and challenge.

## Materials and Methods

### Maternal Hypoxia-Induced IUGR Mouse Model

This study was approved by The University of Western Australia Animal Ethics Committee (approval number RA/3/100/1570). All animals were housed in the Pre-Clinical Facility at The University of Western Australia on a 15:9 light:dark cycle. Thirty pregnant BALB/c mice (gestational day “GD” 7) were obtained from the Animal Resources Center (Murdoch, WA, Australia). Mice were exposed to 10.5% O_2_ from GD 11 to GD 17.5 (hypoxic conditions; IUGR group) (8–12) which corresponds to the pseudoglandular-canalicular stage in fetal mouse lung development, and therefore peak airway development. At GD 17.5, the pregnant mice were removed from the hypoxic chamber and returned to normoxic conditions (21% O_2_) for the remainder of the pregnancy. Another group of pregnant mice remained under normoxic conditions throughout the entire duration of pregnancy (Control group). Only litter sizes of ≤6 pups were included in the study since larger litters reduce body weight independently of maternal hypoxia and compromises milk availability to pups. Offspring were weaned and sexed at 3 weeks of age, with access to standard chow and water *ad libitum*. Weights of offspring were recorded at birth and before lung function assessment (8 weeks of age). A subset of offspring was used to determine the effects of IUGR on diaphragm function and structure in postnatal life (11).

### Allergy Sensitization Protocol

**Figure 1:**
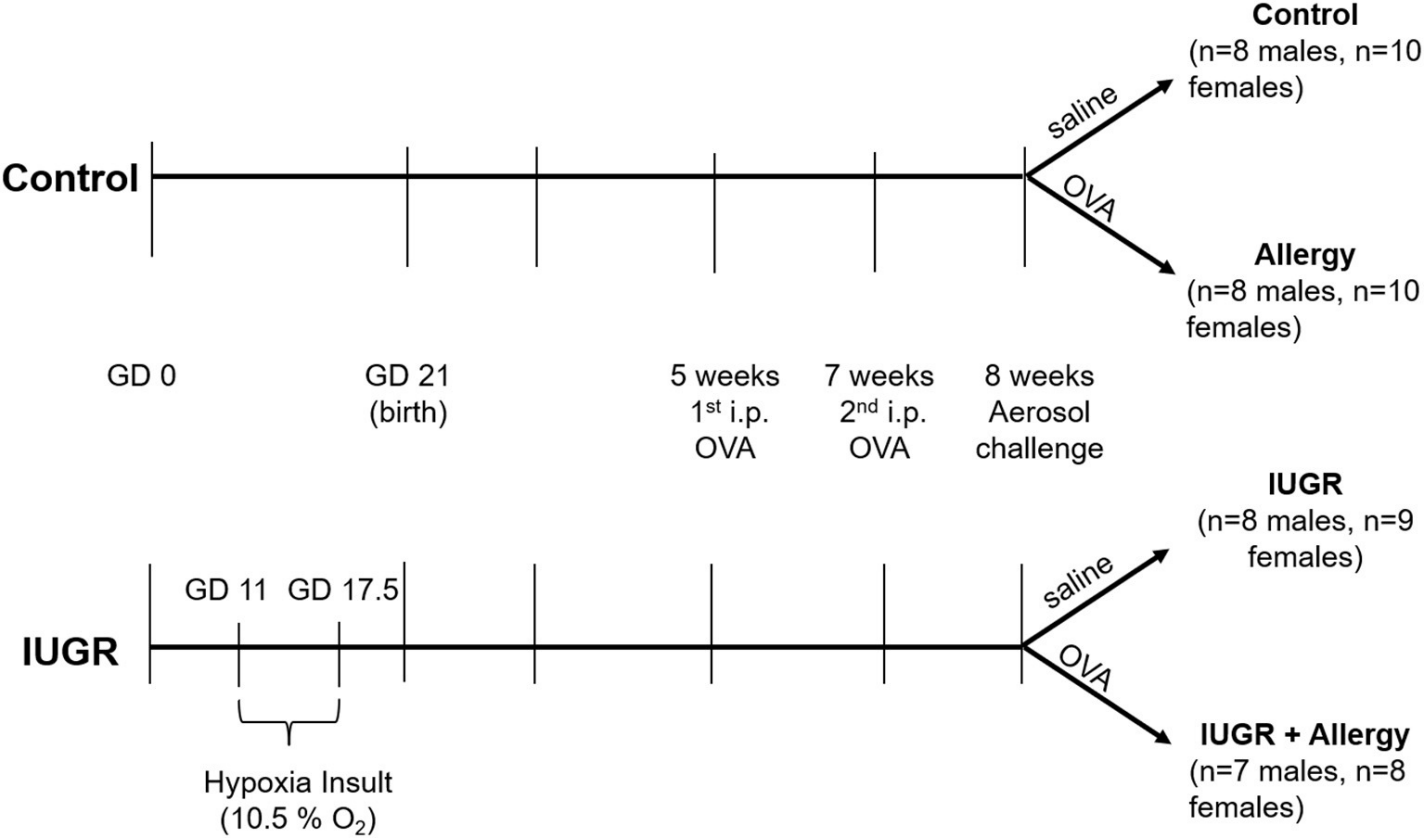
Protocol for IUGR and allergy induction in mice. The superimposition of a maternal hypoxia-induced IUGR model and a well-established allergen protocol, produced 4 experimental groups; Control, Allergy, IUGR and IUGR + Allergy. GD, gestational day; OVA, ovalbumin; i.p., intra-peritoneal; IUGR, intrauterine growth restriction.

An established mouse allergy protocol from our lab was used in this study (13). At 5 and 7 weeks of age, all IUGR and Control offspring received 0.2 mL intra-peritoneal (i.p.) injection containing 5 mg.mL^−1^ of OVA (Sigma, St. Louis, MO, U.S.A.) suspended in 50 mL of alum (Alu-gel-S, Serva, Heidelberg, Germany). At 8 weeks of age, half of the Control and IUGR offspring received 1% OVA aerosol (MPC aerosol medication nebulizer, Braintree Scientific, Inc., MA, U.S.A), whilst remaining offspring received a saline aerosol. This resulted in four experimental groups: Control (males, *n* = 8; females, *n* = 10), normal mice with saline aerosol; Allergy (males, *n* = 8; females, *n* = 10), normal mice with OVA aerosol; IUGR (males, *n* = 8; females, *n* = 9), IUGR mice with saline aerosol; and IUGR + Allergy (males, *n* = 7; females, *n* = 8), IUGR mice with OVA aerosol (Figure 1).

### Lung Function Assessment

Twenty-four h after the aerosol challenge, offspring were anesthetized by i.p. injection of ketamine (0.4 mg.g^−1^ body weight) and xylazine (0.02 mg.g^−1^ body weight). Once under anesthesia, each mouse was tracheostomised, transferred to a FlexiVent system (FX module 1, flexiWare version 7.5, SCIREQ, Montreal, QC, Canada) and then ventilated at 250 breaths.min^−1^ (8, 13). Lung volume history was standardized *via* three slow inflation-deflation manoeuvers up to 20 cmH_2_O transrespiratory pressure.

Respiratory impedance was measured by forced oscillation technique (FOT). Outcomes of airway resistance (R_aw_), tissue damping (G), and tissue elastance (H) were derived from impedance using a constant phase model. Once the mouse was stabilized on the ventilator, FOT measurements were recorded once a min for 5 min to establish baseline respiratory mechanics. Mice were then challenged with 10 s aerosols of saline, followed by increasing doses of methacholine (MCh; β-methacholine chloride, Sigma-Aldrich, St. Louis, U.S.A; 0.1 mg.mL^−1^ to 30 mg.mL^−1^) *via* the Aeroneb ultrasonic nebuliser (SCIREQ). After each challenge, measurements were again recorded every min for 5 min. Peak responses were used for analysis. After final MCh response was measured, 0.1 mL atropine (600 μg/mL) was delivered *via* i.p injection, to reverse airway constriction. Ten min after atropine administration, mice were euthanized with an overdose of ketamine and xylazine (13).

### ELISA Assay

Following euthanasia, blood serum was collected *via* centrifugation of cardiac puncture samples to determine levels of OVA-specific immunoglobulin E (IgE), according to manufacturer's protocol (BioLegend, Inc.) (13).

### Histology and Morphological Analysis

The analysis was only performed in IUGR mice (IUGR compared with IUGR + Allergy groups) since previous study have shown no effect of acute OVA exposure on airway dimensions (13). Lungs were inflation-fixed *in situ* at a transrespiratory pressure of 10 cmH_2_O in 4% formaldehyde (8, 13). The left lung was embedded in paraffin wax and two 5 μm transverse sections were stained with Masson's Trichrome. The first section was acquired just below the transition from extra- to intraparenchymal bronchus [middle region in (14), and the second section marginally deeper into the lung toward the lower region in (14)] (14, 15). All airways within each section were measured. The perimeter of the basement membrane (P_bm_) and areas of the ASM, inner and outer airway wall were measured by Stereo Investigator software (version 10.42.1, MBF Bioscience, United States of America). Airway measurements were averaged within each animal i.e., a case mean was calculated.

### Data Analysis and Statistics

Data were normalized where necessary, to ensure assumptions of parametric tests were satisfied. An unpaired *t*-test was performed to assess differences in birth weight (unsexed) between IUGR and Control offspring. Body weights of offspring at 8 weeks of age were analyzed using a two-way ANOVA, examining the effects of sex and *in utero* treatment.

Lung function data were analyzed using two-way ANOVA, examining the effects of sex in three separate analyses; IUGR effect (Control compared with IUGR group), confirming the effect of IUGR on bronchoconstrictor response in saline exposed offspring only; Allergy effect (Control compared with Allergy group), confirming the effects of allergy on bronchoconstrictor response in Control offspring only; combined effect of IUGR and Allergy (IUGR compared with IUGR + Allergy group), examining the effects of allergy on bronchoconstrictor response in IUGR offspring. Outcomes (R_aw_, G and H) were compared before and after methacholine challenge, and delta change in response to methacholine (e.g., Δ R_aw_, G and H calculated from the difference between 30 mg.mL^−1^ MCh and saline).

Data for OVA IgE levels were analyzed by separate two-way ANOVAs; Control compared with IUGR groups for male and female mice; Control compared with Allergy groups for male and female mice; IUGR compared with IUGR + Allergy groups for male and female mice. Airway dimensions were also analyzed by two-way ANOVA; IUGR and IUGR + Allergy groups for male and female mice. *P*-value < 0.05 was considered statistically significant. Graphical and statistical analysis were conducted using PRISM (version 7, GraphPad Software, La Jolla, CA, U.S.A) and SigmaPlot (version 13, Systat Software, Inc., San Jose, CA, U.S.A).

## Results

### Offspring Growth Outcomes

**Figure 2:**
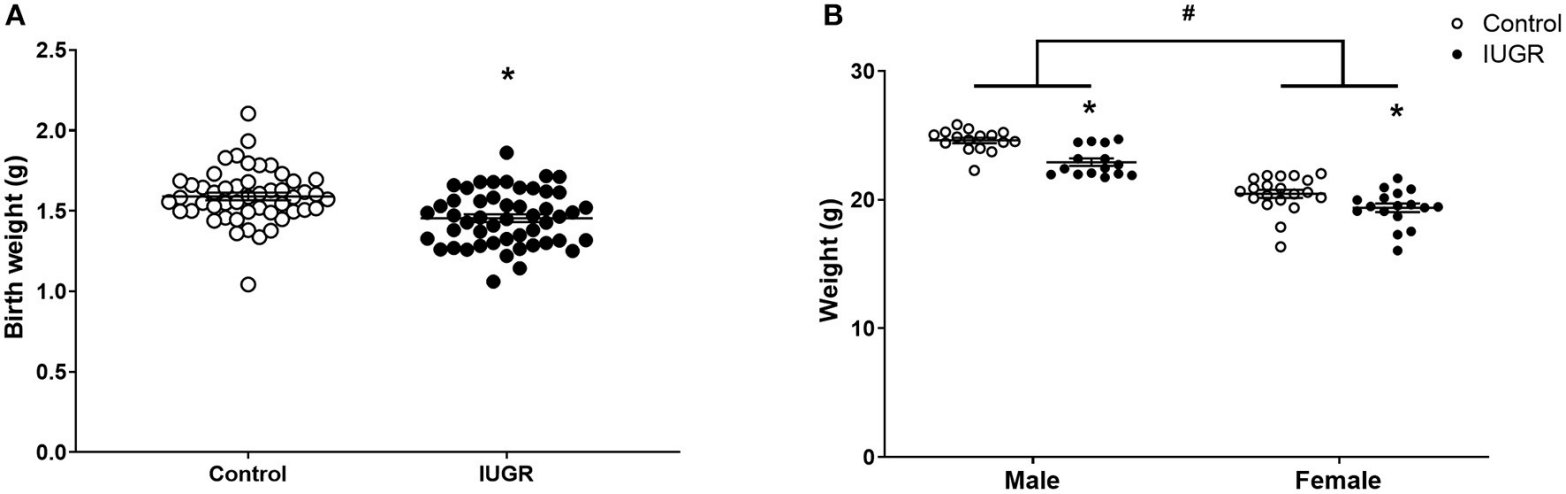
Offspring body weights. Body weights of Control (*n* = 50) and IUGR (*n* = 50) offspring at birth **(A)** and at 8 weeks of age (Control male, *n* = 16; IUGR male, *n* = 15; Control female, *n* = 20; IUGR female, *n* = 17; **B**). Sample size of birth weight is larger than experimental sample size as the birth weight of all pups in each litter was recorded. Data are mean ± SEM. *Significant treatment effect (*P* < 0.05). ^#^Significant sex effect (*P* < 0.05). IUGR, intrauterine growth restriction.

The IUGR offspring were lighter than Control offspring at birth (*P* = 0.0001, unsexed; Figure 2A). The IUGR offspring remained lighter in adulthood (8 weeks; *P* < 0.0001; Figure 2B) and at this age, males were heavier than females (*P* < 0.0001; Figure 2B). There was no effect of OVA exposure on body weight at 8 weeks of age (saline, 21.81 ± 1.79 g; OVA, 21.94 ± 2.05 g; *P* = 0.701).

### Airway Resistance, Tissue Damping, and Elastance

#### IUGR Effect

**Figure 3:**
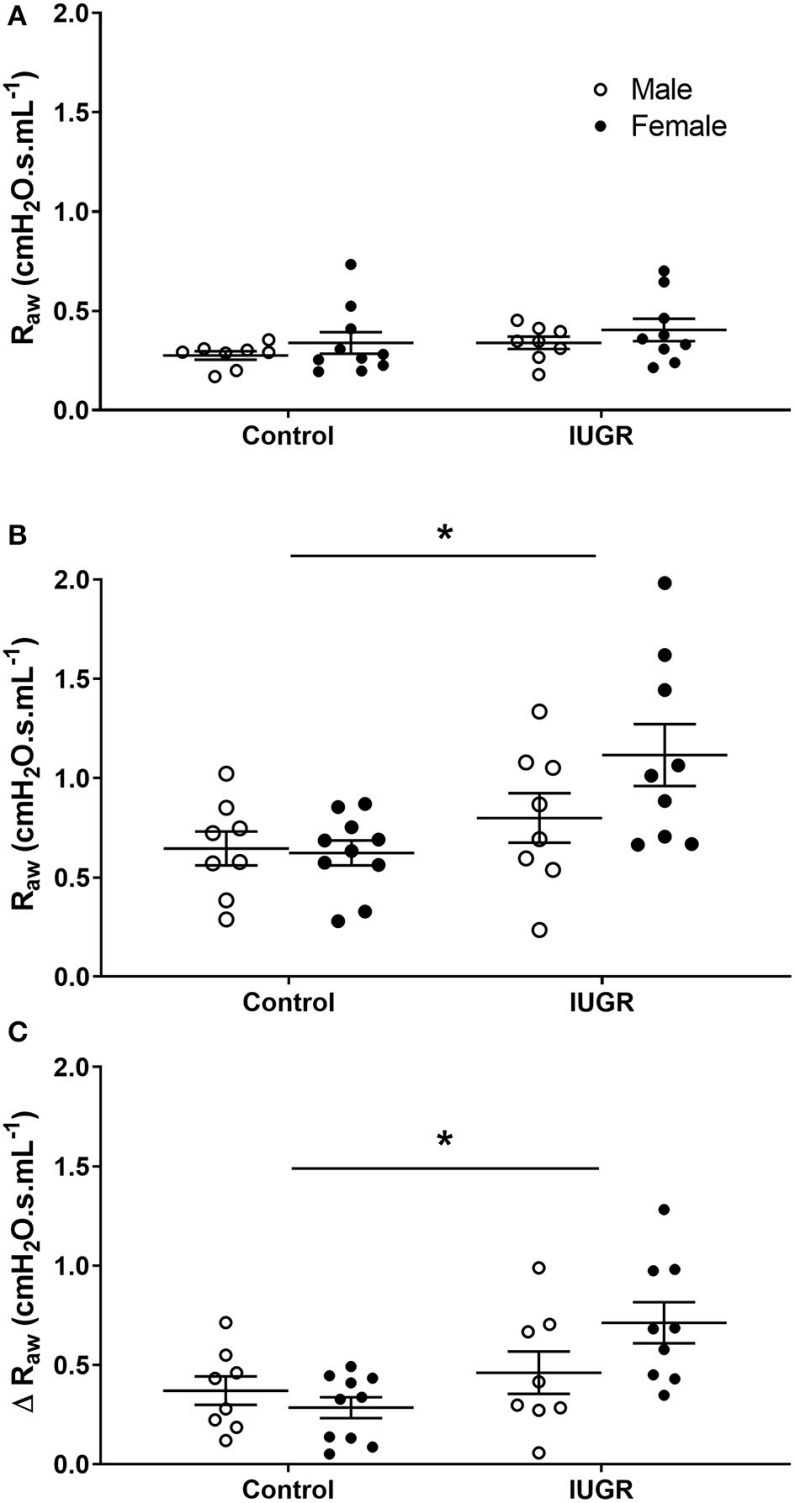
Changes in R_aw_ in Control and IUGR groups. Airway resistance in Control male (*n* = 8) and female (*n* = 10), and IUGR male (*n* = 8) and female (*n* = 9) offspring before **(A)** and after **(B)** MCh challenge, and Δ R_aw_ **(C)**. Data are mean ± SEM. *Significantly different from Control (*P* < 0.05). Males, open circles; Females, closed circles; R_aw_, airway resistance; IUGR, intrauterine growth restriction; MCh, methacholine; Δ, net change after MCh challenge.

To determine the effect of IUGR on bronchoconstrictor response, data were compared in mice exposed only to saline aerosol (i.e., Control and IUGR groups). Before MCh challenge, there was no difference in R_aw_ between Control or IUGR groups (*P* = 0.118) and male or female (*P* = 0.275) mice (Figure 3A). After MCh challenge, R_aw_ (*P* = 0.018; Figure 3B) and Δ R_aw_ (*P* = 0.005; Figure 3C) of IUGR mice were greater than Control. There was no difference in R_aw_ between males and females after MCh challenge (*P* = 0.249).

Before MCh challenge, G (*P* = 0.205; Supplementary Figure 1A) and H (*P* = 0.205; Supplementary Figure 2A) were similar between IUGR and Control groups. After MCh challenge, G (*P* = 0.025; Supplementary Figure 1B) and H (*P* = 0.025; Supplementary Figure 2B) of the IUGR group were greater than the Control group. There was no difference in Δ G (*P* = 0.053; Supplementary Figure 1C) but a greater Δ H in the IUGR mice compared with Control mice (*P* = 0.007; Supplementary Figure 2C). Sex did not affect G (before MCh, *P* = 0.431; after MCh, *P* = 0.429) or H (before MCh, *P* = 0.542; after MCh, *P* = 0.976).

#### Allergy Effect

**Figure 4:**
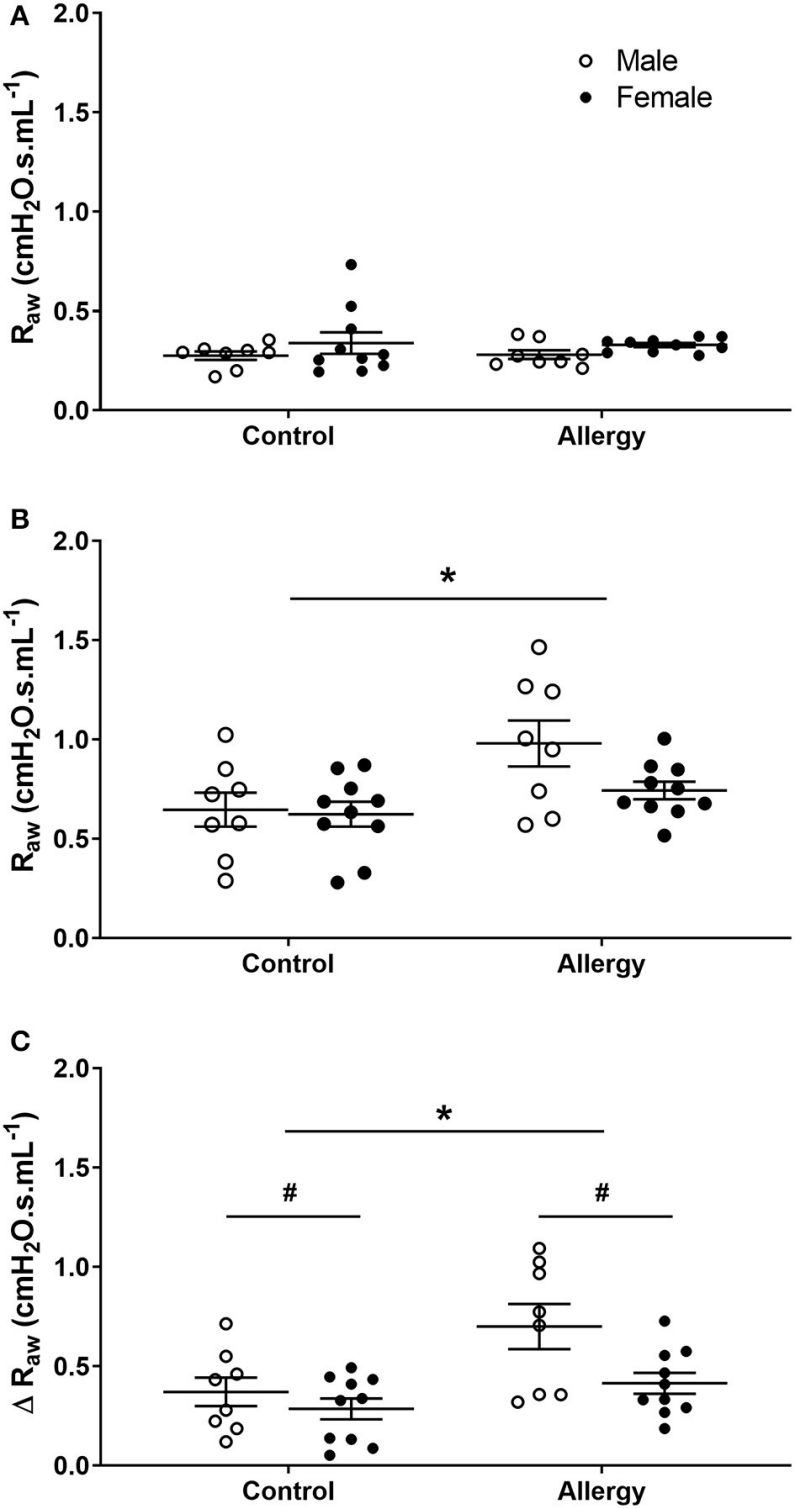
Changes in R_aw_ in Control and Allergy groups. Airway resistance in Control male (*n* = 8) and female (*n* = 10), and Allergy male (*n* = 8) and female (*n* = 10) offspring before **(A)**, and after **(B)** MCh challenge, and Δ R_aw_ **(C)**. Data are mean ± SEM. *Significantly different from Control (*P* < 0.05). ^#^Significantly different from males (*P* < 0.05). Males, open circles; Females, closed circles; R_aw_, airway resistance; MCh, methacholine; Δ, net change after MCh challenge.

Data were compared in Control and Allergy groups to examine OVA effect on R_aw_. Before MCh challenge, there was no difference in R_aw_ between Control or Allergy groups (*P* = 0.671) or between males and females (*P* = 0.109; Figure 4A). Airway resistance (*P* = 0.006; Figure 4B) and Δ R_aw_ (*P* = 0.004; Figure 4C) of the Allergy group was greater than Control group after MCh challenge. Male mice also exhibited a greater Δ R_aw_ compared with females (*P* = 0.016).

Tissue damping of Allergy and Control groups were similar before (*P* = 0.622) and after (*P* = 0.21) MCh challenge (Supplementary Figures 3A,B). Tissue elastance was also similar between Allergy and Control groups before (*P* = 0.837) and after (*P* = 0.065) MCh challenge (Supplementary Figures 4A,B). Sex had no effect on G (before MCh, *P* = 0.758; after MCh, *P* = 0.608) or H (before MCh, *P* = 0.787; after MCh, *P* = 0.89).

#### Combined Effect of IUGR and Allergy

**Figure 5:**
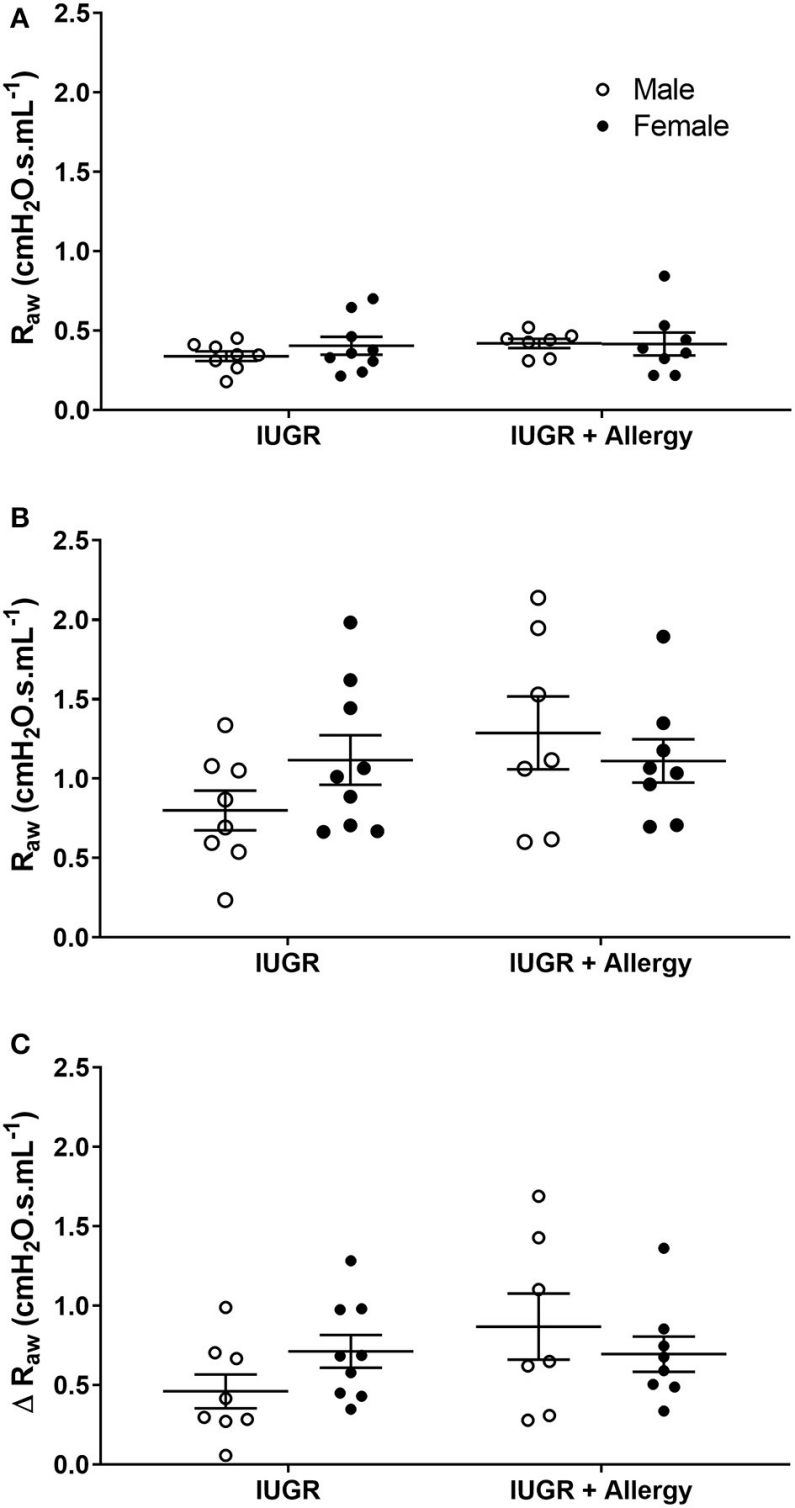
Changes in R_aw_ in IUGR and IUGR + Allergy groups. Airway resistance in IUGR male (*n* = 8) and female (*n* = 9), and IUGR + Allergy male (*n* = 7) and female (*n* = 8) offspring before **(A)** and after **(B)** MCh challenge, and Δ R_aw_ **(C)**. Data are mean ± SEM. Males, open circles; Females, closed circles; R_aw_, airway resistance; IUGR, intrauterine growth restriction; MCh, methacholine; Δ, net change after MCh challenge.

To determine the combined effect of IUGR and Allergy on bronchoconstrictor response, data were compared in IUGR mice that were exposed to either saline or OVA aerosol (i.e., IUGR and IUGR + Allergy groups). There was no difference in R_aw_ of IUGR mice exposed to either saline or OVA, both before (*P* = 0.345; Figure 5A) and after (*P* = 0.149; Figure 5B) MCh challenge, and Δ R_aw_ (*P* = 0.153; Figure 5C). There was also no difference between sexes, before (*P* = 0.841) or after (*P* = 0.670) MCh challenge in R_aw_.

Tissue damping of the IUGR + Allergy group was similar to the IUGR group before (*P* = 0.345) and after (*P* = 0.149) MCh challenge (Supplementary Figures 5A,B). Tissue elastance of the IUGR + Allergy group was also similar to the IUGR group, both before (*P* = 0.578) and after (*P* = 0.225) MCh (Supplementary Figures 6A,B). There was no sex effect on G (before MCh, *P* = 0.62; after MCh, *P* = 0.395) or H (before MCh, *P* = 0.682; after MCh, *P* = 0.932).

#### OVA IgE Levels

Female offspring had higher OVA IgE levels than the male offspring in the IUGR group (*P* = 0.009; Table 1). There were no other differences between groups (*P* > 0.05).

**Table 1:** OVA IgE levels.

	**Male**		**Female**		**Male**		**Female**	
	**Control**	**Allergy**	**Control**	**Allergy**	**IUGR**	**IUGR + Allergy**	**IUGR**	**IUGR + Allergy**
	**(*n* = 8)**	**(*n* = 8)**	**(*n* = 10)**	**(*n* = 10)**	**(*n* = 8)**	**(*n* = 7)**	**(*n* = 9)**	**(*n* = 8)**
OVA IgE (ng/mL)	7.50 ± 2.27	4.87 ± 0.95	6.16 ± 1.13	5.67 ± 0.88	4.98 ± 1.49	3.07 ± 0.65	8.73 ± 1.82	8.33 ± 3.00^#^

*Data are mean ± SEM*.

# *Indicates a significant effect of sex within treatment groups. IgE, immunoglobulin E; IUGR, intrauterine growth restriction; OVA, ovalbumin*.

#### Airway Morphometry of IUGR Offspring

**Figure 6:**
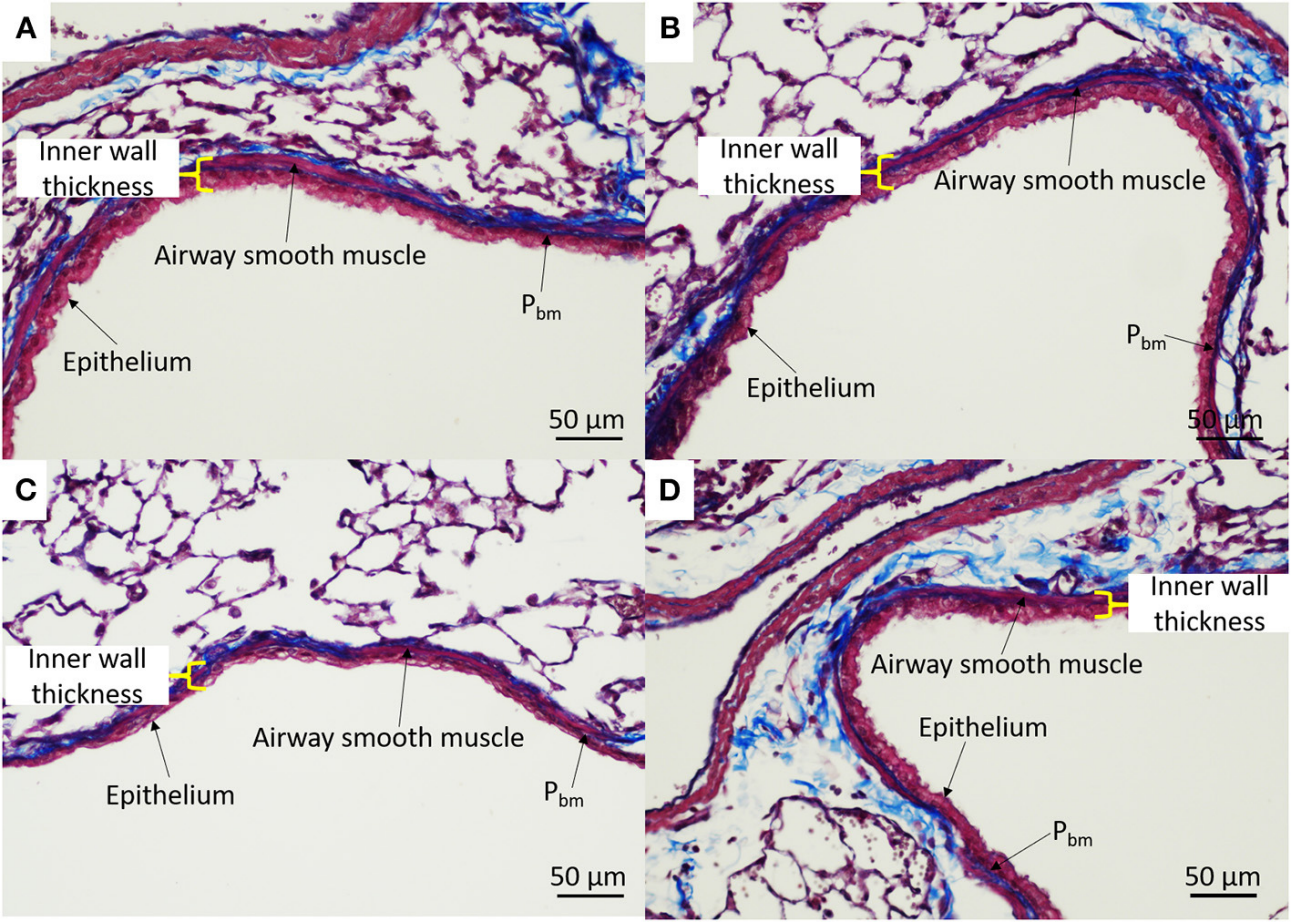
Representative airway histology. IUGR male **(A)**, IUGR + Allergy male **(B)**, IUGR female **(C)**, IUGR + Allergy female **(D)** offspring. P_bm_, perimeter of the basement membrane.

Airway dimensions are provided in Table 2 and representative images are shown in Figure 6. There were no differences in any of the airway wall parameters measured between IUGR and IUGR + Allergy groups (*P* > 0.05).

**Table 2:** Airway dimensions in the IUGR offspring.

	**Male**		**Female**	
**Structure**	**IUGR (*n* = 6)**	**IUGR + Allergy (*n* = 7)**	**IUGR (*n* = 9)**	**IUGR + Allergy (*n* = 8)**
P_bm_ (μm)	1352.38 ± 129.28	1349.12 ± 79.64	1359.06 ± 214.62	1306.91 ± 81.53
Total airway wall (√area/P_bm_)	0.147 ± 0.009	0.154 ± 0.005	0.150 ± 0.004	0.155 ± 0.007
Outer airway wall (√area/P_bm_)	0.069 ± 0.004	0.068 ± 0.002	0.067 ± 0.002	0.069 ± 0.003
Inner airway wall (√area/P_bm_)	0.129 ± 0.008	0.137 ± 0.004	0.134 ± 0.003	0.139 ± 0.006
Epithelium (√area/P_bm_)	0.111 ± 0.006	0.119 ± 0.004	0.121 ± 0.004	0.121 ± 0.005
ASM (√area/P_bm_)	0.056 ± 0.01	0.058 ± 0.002	0.056 ± 0.002	0.056 ± 0.003

*Data are mean ± SEM. The sample size of IUGR male is reduced to 6 as two samples were damaged during tissue collection. ASM, airway smooth muscle; IUGR, intrauterine growth restriction; P_bm_, perimeter of the basement membrane*.

## Discussion

Intrauterine growth restriction and low birth weight are associated with the development of asthma in childhood and adult life (7, 16, 17). Sex and age-dependent changes in AHR within IUGR mice have been documented (8), aligning well with human population studies that show differences in the prevalence of asthma between males and females in early life and adulthood (18–20). Allergy is a major risk factor for asthma (21) and may differentially impact individuals that were growth-restricted *in utero*. The present study used an established mouse model of maternal hypoxia-induced IUGR (8–12) to examine changes in airway responsiveness in IUGR offspring that were subsequently sensitized and challenged with an allergic stimulus i.e., OVA. Findings were unexpected in that the independent effects of IUGR and allergy in promoting AHR did not enhance bronchoconstriction when both abnormalities were combined.

The animal model of IUGR used in this study is robust; the reduction in body weight (at birth and 8 weeks of age) is comparable to that observed previously (8–12), affecting both male and female offspring (10, 11). An acute allergy exposure protocol was preferred as this produces an inflammatory-mediated increase in airway responsiveness (13, 22), without altering body weight (13, 23). Airway responsiveness was assessed from changes in resistance measured using FOT which showed an exaggerated response to MCh after OVA, consistent with AHR. An exaggerated increase in H was also observed, as has been previously documented (13, 22), although G was unchanged. Tissue damping response is at times affected by OVA (22), varying with aerosol deposition patterns (24).

The IUGR and OVA protocols have revealed several interesting biological phenomena, the first of which is apparent after pure sensitization, even prior to bronchial challenge. As discussed, IUGR offspring in the absence of sensitization exhibit sex-dependent changes in airway responsiveness (8). While adult female offspring are hyperresponsive after IUGR (8), males are hyporesponsive, an effect that may well be explained by reduced contractile capacity of the ASM layer (9). We here now observe that after OVA sensitization, male and female mice are both hyperresponsive, in that males switched from a hypo- to hyperresponsive phenotype, manifested by an exaggerated increase in resistance, damping and elastance. Why allergic sensitization modifies airway responsiveness in male offspring is unclear. It has been previously shown that IgE response to house dust mite and OVA sensitization in male and female placentally restricted lambs is increased compared with Control lambs (25), but the OVA-specific IgE levels were comparable between our male and female Control and IUGR mice. One possibility is that a normal response to OVA interacts with a primed basal immunity, previously documented in the offspring of male IUGR mice, specifically elevated IL-13 in BAL fluid (10). Interleukin-13 is produced by natural killer cells after OVA sensitization and is strongly correlated with the Th2-induced allergic cascade and exaggerated bronchoconstrictor response (26).

The primary aim of the study was to examine how a prenatal insult (IUGR) interacts with a common risk factor for asthma i.e., aeroallergen. It seemed reasonable to hypothesize that if IUGR and aeroallergen independently increase airway responsiveness, then combined the respiratory abnormality would worsen. Indeed, aeroallergen exaggerates bronchoconstriction in a mouse model of ASM thickening caused by localized expression of a growth factor (13). Results showed no additional effect (additive or synergistic) of IUGR and allergy on airway responsiveness. Previous studies examining the relationship between atopy and low birth weight report an attenuated allergic response in IUGR individuals (27–29), including a reduced incidence of atopic dermatitis and food allergies (29). The relationship between low birth weight and asthma in childhood and adult life (7, 16, 17) may therefore reflect innate changes in airway responsiveness rather than a tendency to allergic disease.

We have previously reported no change in postnatal airway morphology after IUGR (8, 12) or acute exposure to OVA (13), including P_bm_ and the thickness of ASM and epithelial layers, and total airway wall thickness. In this study, we investigated whether the effects of IUGR and allergy interact to produce airway remodeling. Airway wall structure after IUGR was not affected by subsequent exposure to OVA. These data suggest that the increased bronchoconstrictor response observed in IUGR and OVA-induced allergic mice is independent of any change to airway wall structure.

A commonality between changes elicited by IUGR and OVA is airway inflammation; the former leading to increased lung macrophages at 2 and 8 weeks in mice (10) and in adult rats (30). The effects of macrophages are varied in asthma, exerting regulation through phagocytic, anti- and pro-inflammatory activities (31). It is conceivable that macrophage infiltration after IUGR favors excessive bronchoconstriction; macrophages are developed *in utero* and self-maintained throughout life *via* proliferation (32), and are activated by IL-13 (33) which appears in greater concentrations in IUGR male offspring (10). In patients with chronic obstructive pulmonary disease, a treatment-driven reduction in AHR was independently associated with a reduction in sputum macrophages as well as lymphocytes (34). At the same time, if anti-proliferative effects of macrophages dominate, an additional effect of IUGR could be to resist further abnormality following exposure to aeroallergen. Future research on how IUGR alters macrophage behavior is therefore warranted.

Other studies have also queried potential interactions between IUGR and allergen exposure. Landgraf et al. (35) used a gestational maternal undernutrition rat model of IUGR and reported an increase in airway responsiveness after OVA sensitization and challenge. However, in the study by Landgraf et al., IUGR alone did not appear to increase bronchoconstrictor response, although the method of assessing constriction from changes in perfusion pressure of excised lungs is quite indirect and may lack sensitivity (35). Two other studies which also used a maternal undernutrition-induced IUGR model in mice and rats demonstrate increased lung inflammation in IUGR offspring following OVA sensitization and challenge (36, 37). Differences in model parameters likely contribute to these disparate findings, including the method used to induce IUGR (hypoxia or undernutrition), duration of exposure and species. Despite these differences, animal models of IUGR seem to consistently report changes in airway biology that are of relevance to asthma.

We acknowledge that there are differences in mouse lung anatomy compared with human (38) which to some extent reduces the model's relevance to disease. However, the data generated allows us to form hypotheses on how prenatal and postnatal disorders interact and reveals new avenues for therapy. Airway responsiveness is modified by numerous exposures that do not necessarily share the same underlining mechanism. Given that airway biology can be modified by such a diverse range of factors, this could explain why not all treatments have the same efficacy on a given asthmatic patient. In addition to postnatal preventative measures for asthma, the prenatal window of susceptibility needs further consideration.

In summary, results indicate that while sensitized IUGR offspring are hyperresponsive to an inhaled bronchoconstrictor agonist, aeroallergen does not cause further functional disruption. These findings suggest that innate changes in bronchial reactivity are more likely to explain associations between IUGR and asthma, rather than an allergen driven inflammatory response within the lungs.

## Data Availability Statement

The original contributions presented in the study are included in the article/Supplementary Material, further inquiries can be directed to the corresponding author.

## Ethics Statement

The animal study was reviewed and approved by The University of Western Australia Animal Ethics Committee (approval number RA/3/100/1570).

## Author Contributions

KW and PN: conceived and designed the experiments. JK, CW, and KW: performed the experiments. All authors analyzed the data, drafted and helped critically revise, read and approve the manuscript.

## Conflict of Interest

The authors declare that the research was conducted in the absence of any commercial or financial relationships that could be construed as a potential conflict of interest.
