# Supplementary material for: Intrauterine Growth Restriction Promotes Postnatal Airway Hyperresponsiveness Independent of Allergic Disease
Source: Front Med (Lausanne). 2021 May 31;8:674324. doi: 10.3389/fmed.2021.674324 (PMC8200568; doi:10.3389/fmed.2021.674324)
Supplement: Supplementary file 1 [file Data_Sheet_1.docx]

Supplementary data

# Figure captions

**Supplementary Figure 1. Changes in G in Control and IUGR groups.** Tissue damping in Control male (n=8) and female (n=10), and IUGR male (n=8) and female (n=9) offspring before (A) and after (B) MCh challenge, and Δ G (C). Data are mean ± SEM. *Significantly different from Control (*P*<0.05). Males, open circles; Females, closed circles; G, tissue damping; IUGR, intrauterine growth restriction; MCh, methacholine; Δ, net change after MCh challenge.

**Supplementary Figure 2. Changes in H in Control and IUGR groups.** Tissue elastance in Control male (n=8) and female (n=10), and IUGR male (n=8) and female (n=9) offspring before (A) and after (B) MCh challenge, and Δ H (C). Data are mean ± SEM. *Significantly different from Control (*P*<0.05). Males, open circles; Females, closed circles; H, tissue elastance; IUGR, intrauterine growth restriction; MCh, methacholine; Δ, net change after MCh challenge.

**Supplementary Figure 3. Changes in G in Control and Allergy groups.** Tissue damping in Control male (n=8) and female (n=10), and Allergy male (n=8) and female (n=10) offspring before (A) and after (B) MCh challenge, and Δ G (C). Data are mean ± SEM. Males, open circles; Females, closed circles; G, tissue damping; IUGR, intrauterine growth restriction; MCh, methacholine; Δ, net change after MCh challenge.

**Supplementary Figure 4. Changes in H in Control and Allergy groups.** Tissue elastance in Control male (n=8) and female (n=10), and Allergy male (n=8) and female (n=10) offspring before (A) and after (B) MCh challenge, and Δ H (C). Data are mean ± SEM. *Significantly different from Control (*P*<0.05). Males, open circles; Females, closed circles; H, tissue elastance; IUGR, intrauterine growth restriction; MCh, methacholine; Δ, net change after MCh challenge.

**Supplementary Figure 5. Changes in G in IUGR and IUGR + Allergy groups.** Tissue damping in IUGR male (n=8) and female (n=9), and IUGR + Allergy male (n=7) and female (n=8) offspring before (A) and after (B) MCh challenge, and Δ G (C). Data are mean ± SEM. Males, open circles; Females, closed circles; G, tissue damping; IUGR, intrauterine growth restriction; MCh, methacholine; Δ, net change after MCh challenge.

**Supplementary Figure 6. Changes in H in IUGR and IUGR + Allergy groups.** Tissue elastance in IUGR male (n=8) and female (n=9), and IUGR + Allergy male (n=7) and female (n=8) offspring before (A) and after (B) MCh challenge, and Δ H (C). Data are mean ± SEM. Males, open circles; Females, closed circles; H, tissue elastance; IUGR, intrauterine growth restriction; MCh, methacholine; Δ, net change after MCh challenge.

# Figures


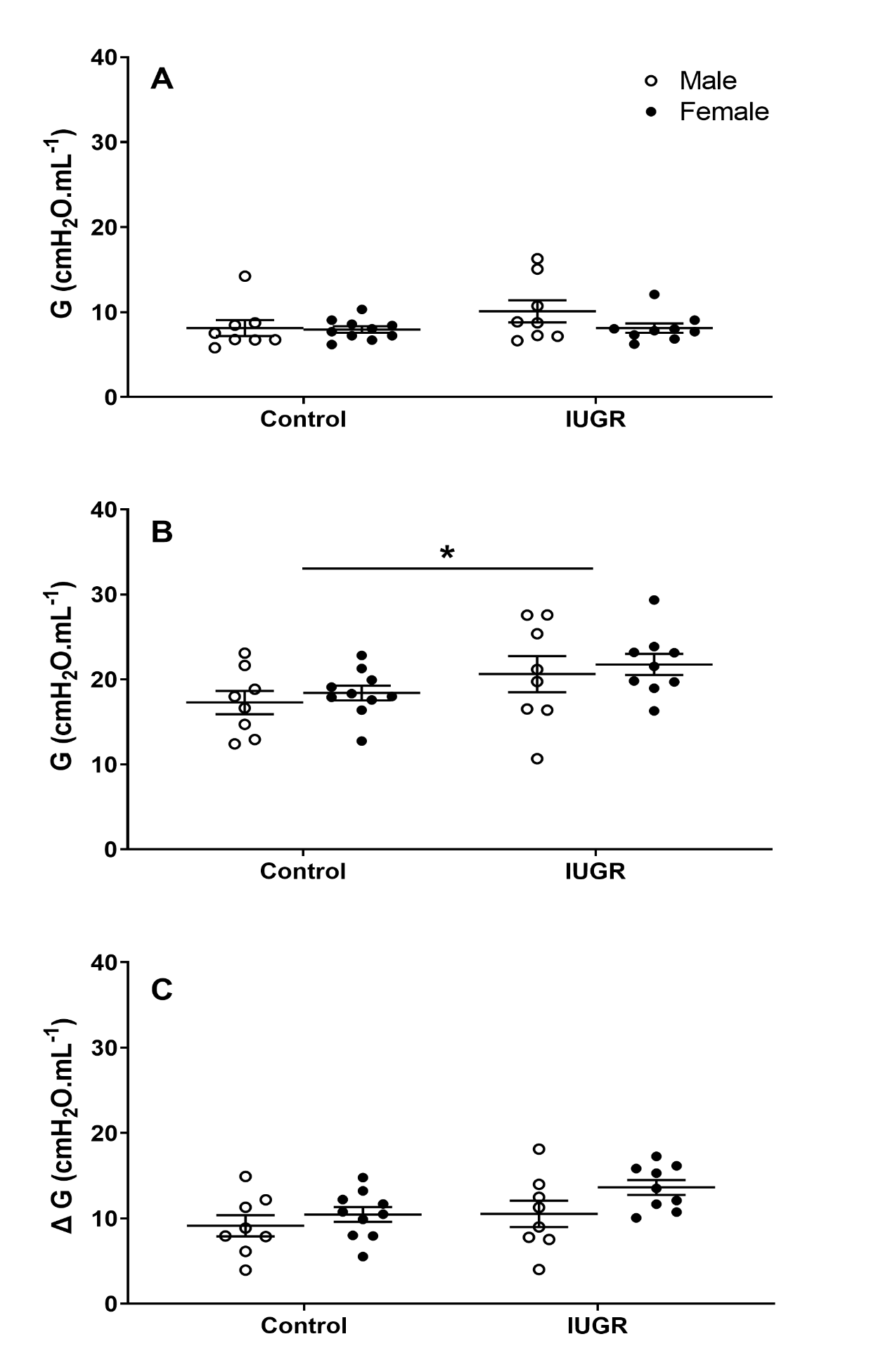
Supplementary Figure 1.


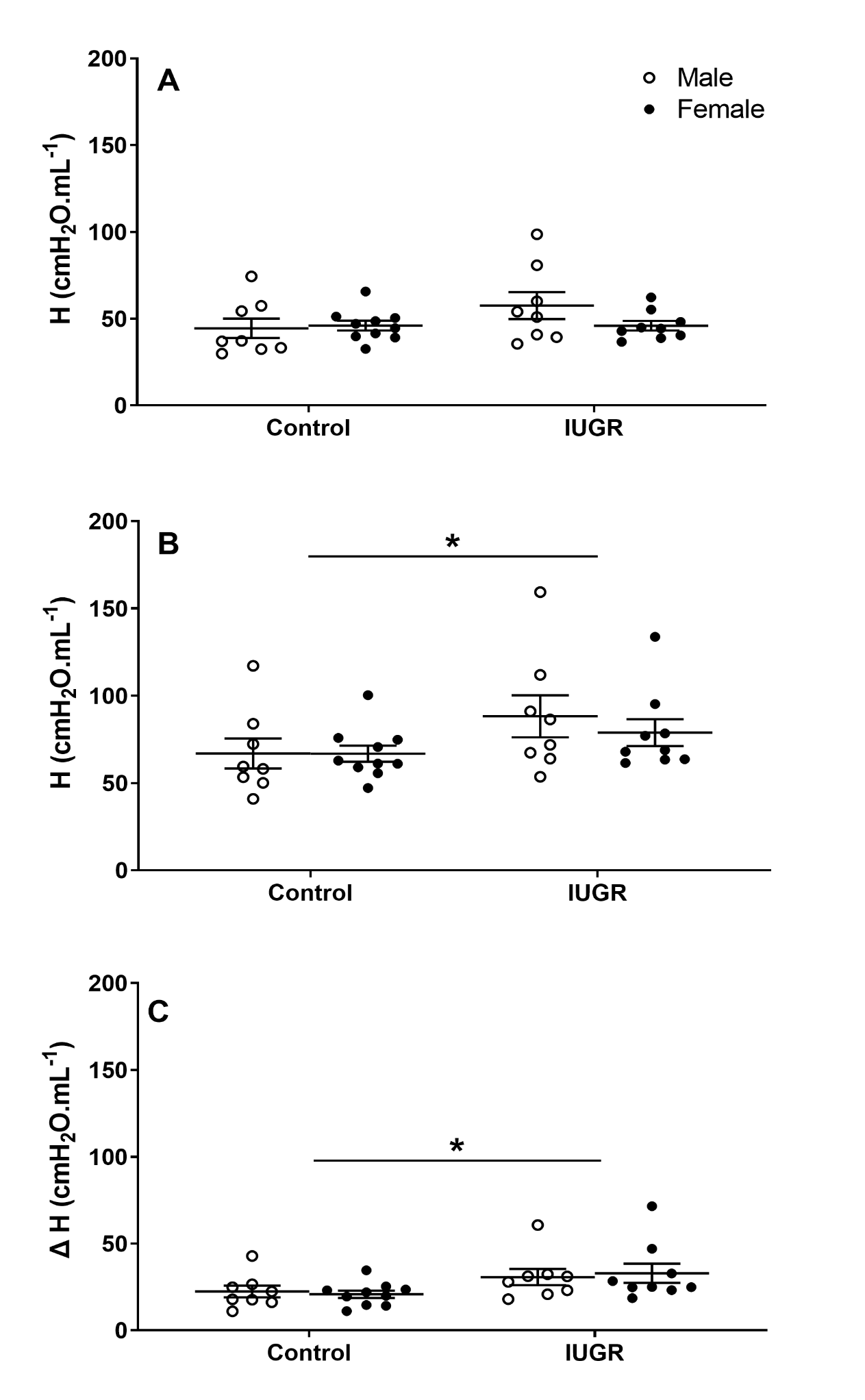


Supplementary Figure 2.


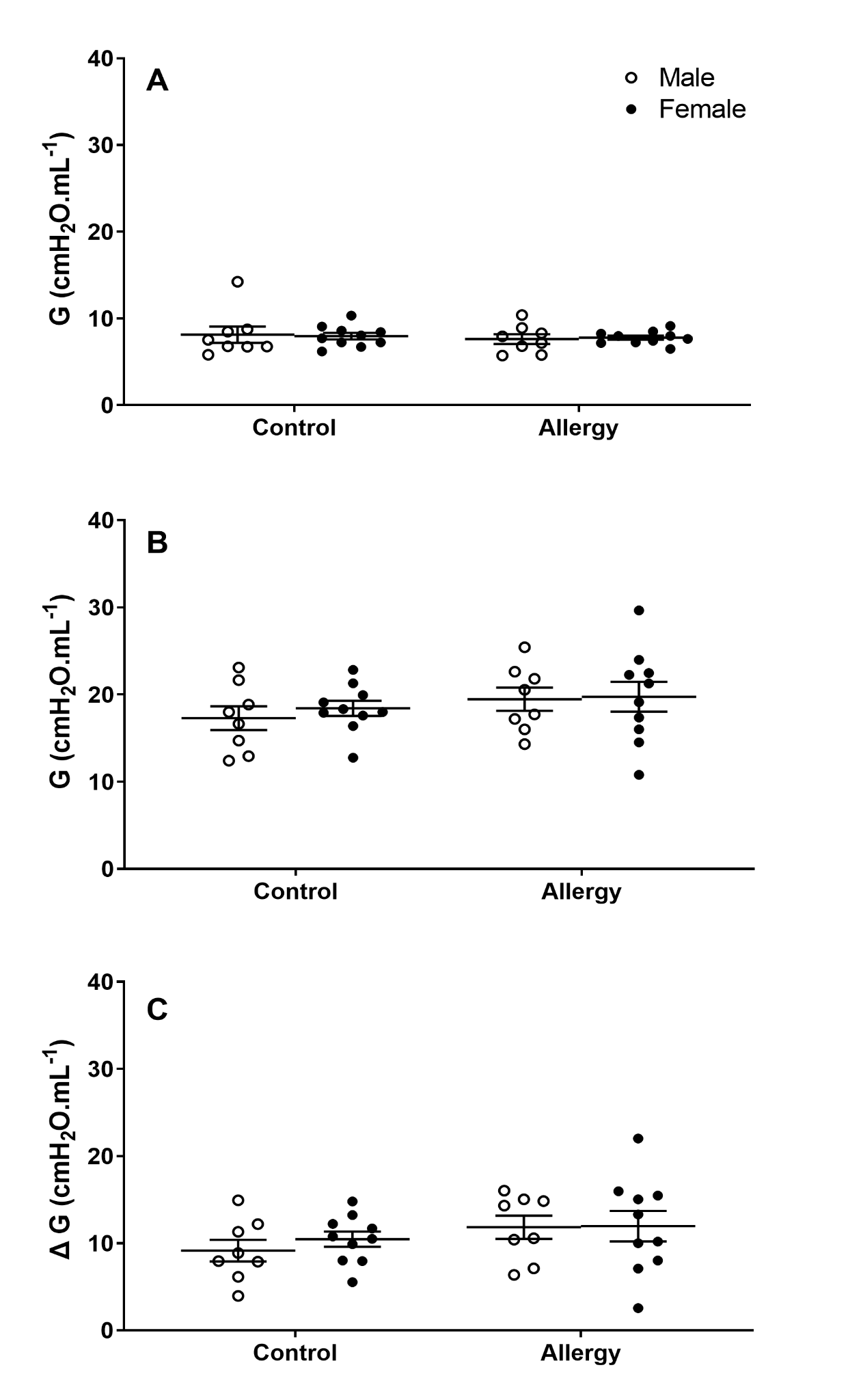
 Supplementary Figure 3.


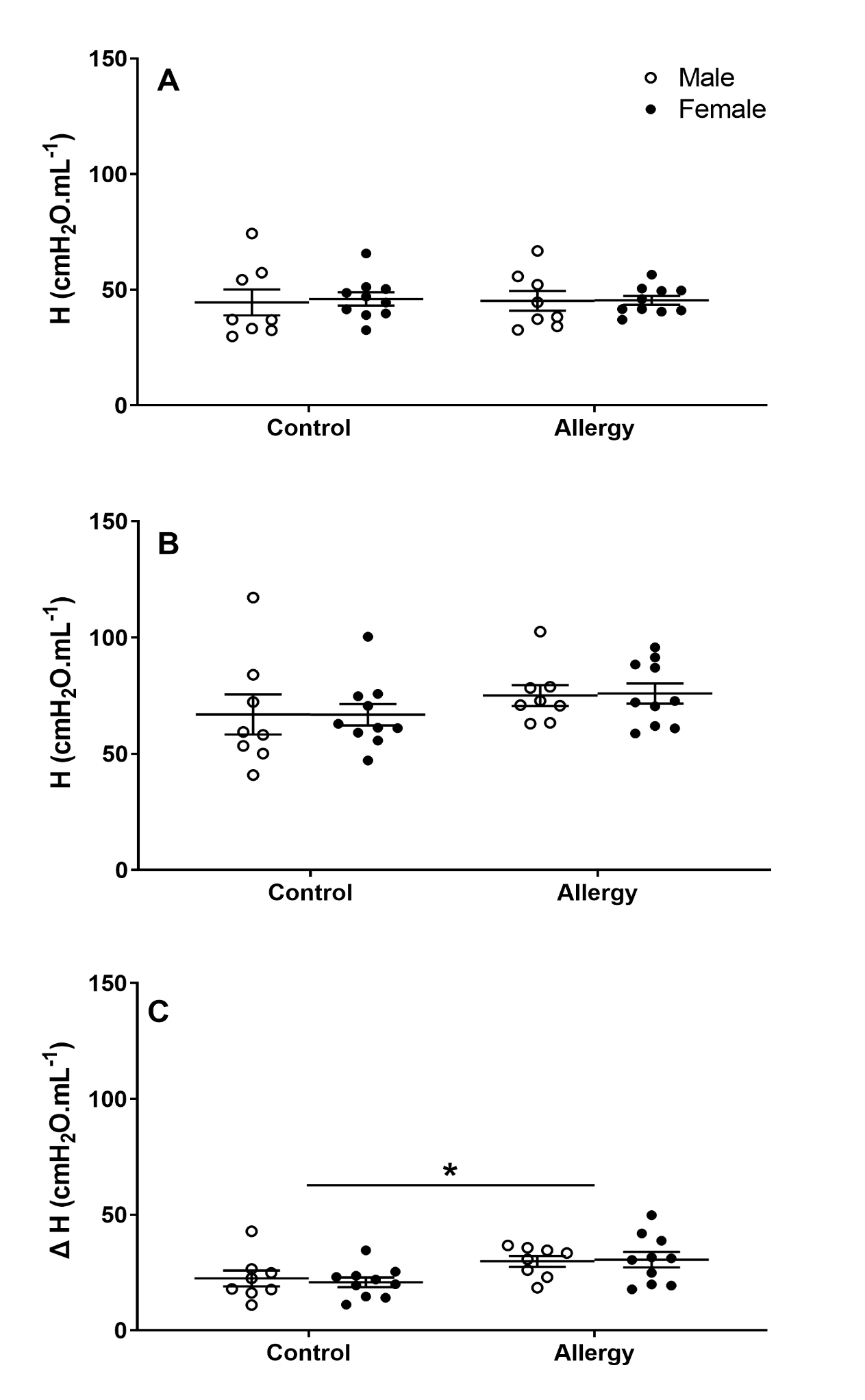


Supplementary Figure 4.


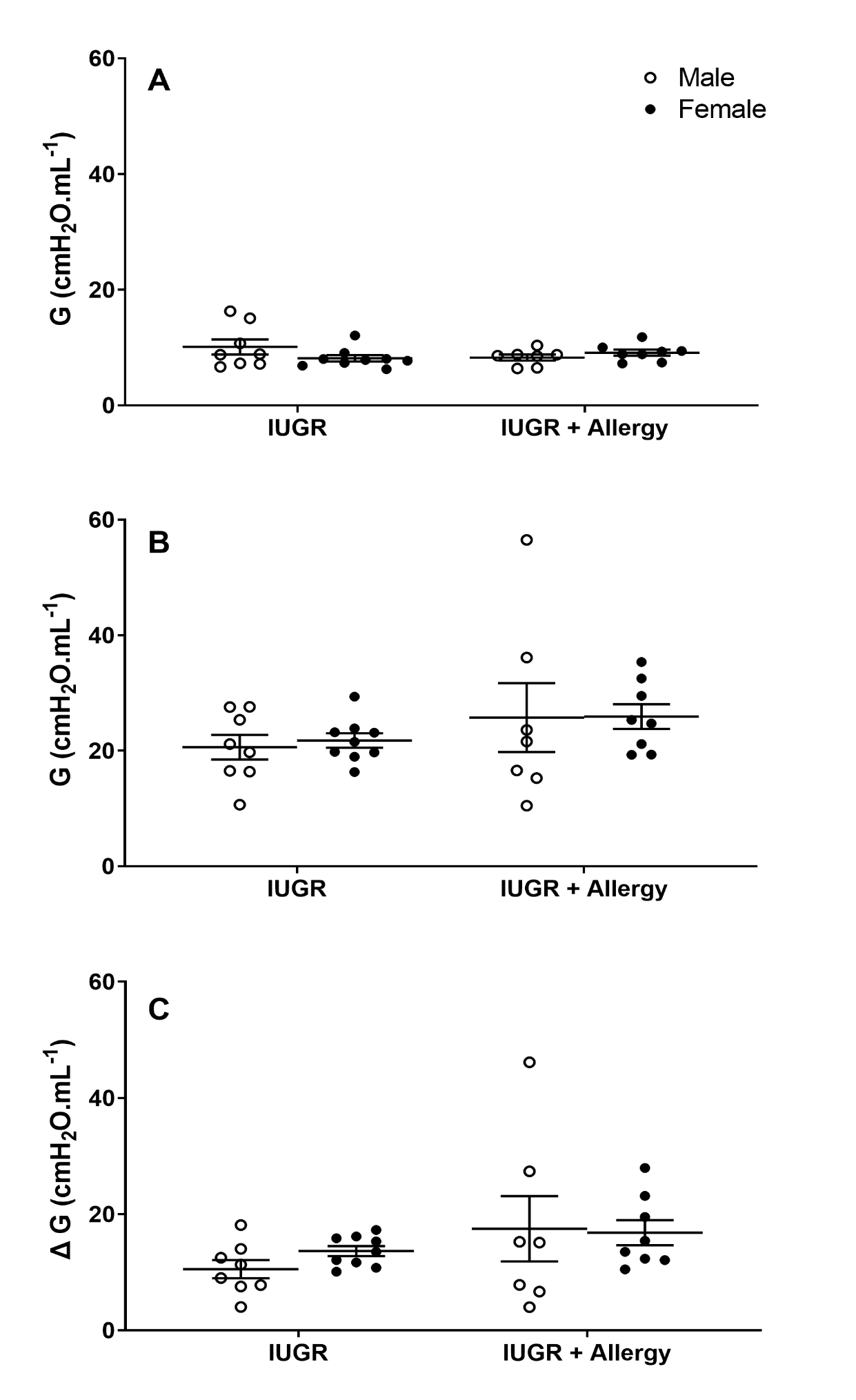


Supplementary Figure 5.


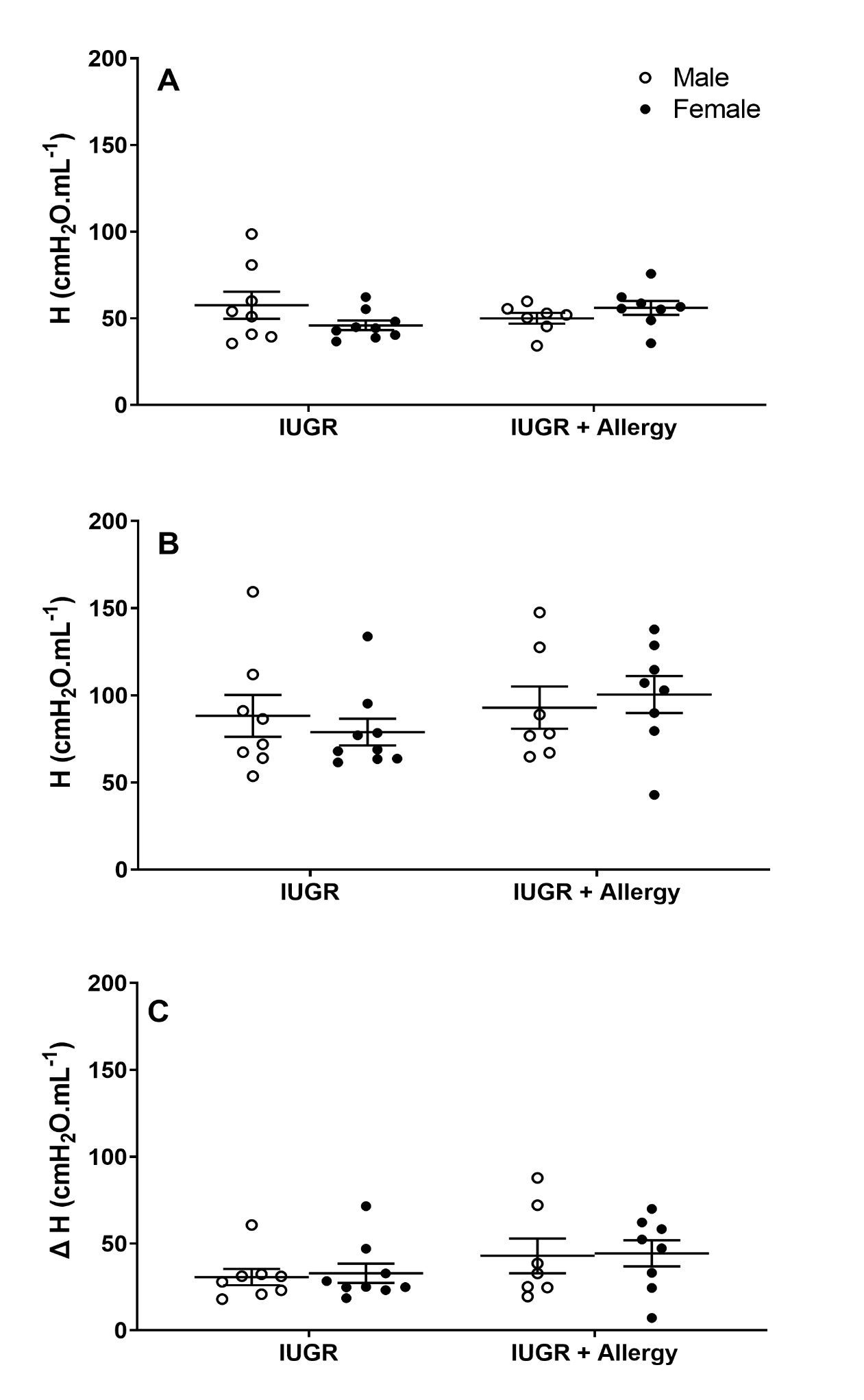


Supplementary Figure 6.
